# Creativity in public involvement: supporting authentic collaboration and inclusive research with seldom heard voices

**DOI:** 10.1186/s40900-021-00260-7

**Published:** 2021-03-17

**Authors:** Katherine Broomfield, Claire Craig, Sarah Smith, Georgina Jones, Simon Judge, Karen Sage

**Affiliations:** 1 Gloucestershire Health and Care NHS Foundation Trust, Gloucestershire, UK; 2 Manchester Metropolitan University, Manchester, UK; 3 Bristol Speech and Language Therapy Research Unit, Steps and Pines, Southmead Hospital, Westbury on Trym, Bristol, BS10 5NB UK; 4 Sheffield Hallam University, Sheffield, UK; 5 Leeds Beckett University, Leeds, UK; 6 Barnsley Hospitals NHS Foundation Trust, Barnsley, UK; 7 University of Sheffield, Sheffield, UK

**Keywords:** Patient-reported outcome measure, PROM, Communication difficulty, Augmentative and alternative communication, AAC, Public involvement, PI

## Abstract

**Background:**

The role of public involvement (PI) in healthcare research is growing in importance and it is imperative that researchers continuously reflect on how to promote the inclusion of patients and service users in the design and delivery of research. PI offers a mechanism for end-users to be involved planning, executing, and reporting research. Some patient groups, including people who have communication difficulties, may struggle to engage in the methods traditionally employed to promote PI engagement such as questionnaires and focus groups.

**Methods:**

This article describes a longitudinal case-study of a PI group, consisting of people who have communication difficulties, for a patient-reported outcome development project. Creative methods, informed by the participatory design principles of enacting, seeing and doing, were introduced stepwise into seven PI meetings. Data from video and visual minutes were used to evaluate the impact of the methods, following each group. Feedback, in the form of verbal and visual outputs taken directly from group meeting minutes, along with vignettes evidenced the impact of the methods on the project and group members.

**Results:**

Creative methods enabled the PI group members to successfully contribute in meetings, to interact dynamically and to engage with the aims and processes of the research project. Their involvement facilitated the development of accessible recruitment materials, informed data analysis and supported the dissemination of project outputs. Employing creative methods also enabled both PI group members and the academic team to reflect on their own roles within the research project and the impact that their active involvement in the PI group has had on their personal development and perspectives on research.

**Conclusion:**

The impact of using creative methods in PI for this patient-reported outcome measure (PROM) development project improved collaboration and understanding between PI members and the academic team. The authentic engagement of people who have communication difficulties in PI generated a more accessible project in terms of both process and impact. Creativity has applicability beyond people whose communication is non-verbal; it should be harnessed by research teams to identify and breakdown barriers to involvement to develop outcome tools that reflect the diversity of our populations.

## Plain English summary

Public involvement (PI) is the term used to describe the role of members of the public in research projects. People carrying out research about healthcare realise that involving members of the public is important; it can help researchers to plan and prepare projects so that the way research is carried out and reported makes sense to patients and ensures that we research important questions. Public involvement typically uses group meetings and discussions which can be difficult for people who have communication difficulties.

This article describes a process for introducing creative activities that was used in a PI group for people who have communication difficulties. The activities included using videos, pictures and objects to support group members to be more involved in meetings. The research team noted examples of when these activities were successful and gathered feedback about them from group members.

Using activities supported people with communication difficulties to interact with one another in PI meetings. The activities helped them to understand the words and processes that are used in research so that they could help to make these aspects of the project simpler for others. They also discovered how their own skills and experience fit into the project.

Thinking creatively and using activities rather than just words and talking helped to establish a successful public involvement group with people who have communication difficulties. The group has supported the research team to create a more accessible research project. Using these types of activities may be a helpful way to involve other people who find groups and discussion difficult in research.

## Background

The past few decades have witnessed an increase in public demand for transparency, accountability and emancipation of decision-making across a range of public services [1]. The UK government responded by investing in both policy and legislation that requires greater public involvement in health and social care [2, 3], and financially in initiatives like the UK Standards for Public Involvement [4] and in organizations which promote and support public involvement, such as INVOLVE. The UK Standards for Public Involvement were the result of a collaborative effort by key stakeholders to describe what good public involvement looks like in health and social care research. INVOLVE is a publicly funded organization in the UK whose aim is to promote patient and public involvement in research. The term public involvement (PI) refers to the role that patients and service users can play at different stages in the research process in advising and guiding decision-making, including (though not exclusively): question formation, study design, conduct, and governance [5]. INVOLVE [6] define ‘involvement’ as: “where members of the public are actively involved in research projects …” (2012, page 7), and ‘collaboration’ as: “an ongoing partnership [ …] where decisions about the research project are shared” (2012, page 21).

A patient reported outcome (PRO) is a measurement that has come directly from a patient about their health status, without interpretation by a clinician or anyone else [7]. A patient-reported outcome-measure (PROM) is the instrument or tool used to collect this information. The use of patient-reported outcome measures (PROMs) can help to empower patients and foreground their values in healthcare interactions [8]. The development of a PROM tool requires an iterative set of rigorous research processes design to a) identify the relevant concepts relating to the health condition being measured, b) assess their acceptability to end-users and c) evaluate of the validity of the tool [7]. Patient input is essential throughout the PROM development process if the final tool is to reflect the priorities of the population with whom it is to be used. A recent review highlighted that only 6.7% of PROM development studies had public involvement at every stage [9]. In response Carlton et al. [10] drew up a framework for fully incorporating PI in PROMs (Table 1) and this is used as a guide within the work presented here.

**Table 1:** Public Involvement Framework for PROM Development; Adapted from Carlton et al. [10]

PROM development stage	PI involvement
**1. Establish a need for new or refined PROM**	- Review existing PROMs - Critique existing PROMs - Determine whether a new PROM is needed
**2. Development of a conceptual framework**	- Review of conceptual model to ensure validity
**3. Identifying item content**	- Input on study design - Input on culturally appropriate issues - Input on participant-facing documents - Input on ethics and governance considerations
**4. Item development**	- Analysis and interpretation of qualitative interviews - Advice and input on working of potential items
**5. Item reduction**	- Identify potentially redundant items - Identify items that could benefit from rewording - Input and advice in ordering of items
**6. Pre-testing of items (cognitive interviews/debriefing)**	- Input on study design, methodology, recruitment, design and content of public facing document, and conducting the interviews - Analysis and/or interpret results
**7. Psychometric survey design**	- Input on study design
**8. Psychometric survey analysis**	- Advice on the interpretation of the results - Ensure validity of findings
**9. Selection of items for the PROM**	- Advice on final selection of items - Consideration of number of items to be included - Advice and input into how PROM may be used in clinical settings
**10. Design of the PROM**	- Advice and input of format and layout of PROM - Advice on instructions of how to complete PROM, framing of questions, working of response options, and order of items
**11. Dissemination and promotion of PROM**	- Co-authorship and co-presenting - Advice on strategies for wider dissemination - Input on content of materials to ensure appropriate language and terminology are used

How we capture patient perspectives, i.e. who we ask, what we ask, and how we ask it, are necessary considerations if the PROM under development is to be useful and acceptable to the population of end-users. PI can be a means by which research teams can incorporate patient perspectives in the design and process of conducting PROMs research [11] and to embed the patient voice in the final measure. PI ensures that information, methods, and analysis at each stage of the PROM development cycle are considered with the end-users in mind. PI runs the risk of being tokenistic if not carried out thoughtfully or if the methods we use are insensitive to the needs of specific populations [12]. For example, PI has tended to rely on discussion groups, interviews, and questionnaires to gather expert opinion. Yet there are many patient groups for whom such conventional methods of engagement are not accessible, such as those who have communication difficulties.

People may have communication difficulties as a result of conditions from birth such as cerebral palsy and autism; or may acquire communication difficulties as adults following stroke, cancer or as a result of degenerative conditions such as Motor Neuron Disease/Amyotrophic Lateral Sclerosis (MND/ALS). Approximately 300,000 people in the UK have complex communication difficulties that result in the need to have support with their communication [13]. Such support may include a set of strategies known as augmentative and alternative communication (AAC), which can help people with communication difficulties to convey their message. AAC include tools ranging from paper-based systems such as picture books to electronic or computer-based devices that transform messages inputted into synthetic speech output [14]. Traditional speech- or language-dependent engagement methods, such as focus groups, can be exceptionally challenging for people who use AAC and can inhibit their involvement in PI.

Avoiding tokenism requires collaboration and establishing an arena and a set of methods that facilitate participation and the building of positive relationships which enable people to express opinions and critique decisions [12]. Establishing a set of suitable methods demands that research teams attend to the particular needs and challenges of the people who will form the PI group. This has been achieved in two previous studies, one with people with aphasia and another with people using AAC. People who have aphasia following stroke (a difficulty with understanding and using spoken and/or written language) have been included in PI for research through careful facilitation of their communication needs by scaffolding meetings with supportive keywords, pictures and gestures [15]. Two people who have communication difficulties and use AAC were recently involved in a research project as co-researchers [16]. The co-researchers were facilitated to participate in the research by accessing training and support from other members of the research team. Although the authors acknowledge that co-researchers were of great value to the project overall, they identified that the costs and time associated with providing such intense support were significant [16]. People who use AAC are not a homogenous group and frequently experience co-existing and complex challenges to involvement in addition to communication difficulties such as cognitive, physical, and visual disabilities [13]. Collaborating with people who use AAC in PI cannot rely on one particular set of strategies and may require additional time, thought and resource from the academic research team to enable their involvement. In order to harness a broad range of perspectives, a PI group who use different AAC systems and who have a range of communication difficulties may provide more diversity of opinion and bring different life experiences to the table.

When methods traditionally used by health researchers fall short of being appropriate for specific groups of people or questions, exploring the techniques from other disciplines can provide solutions. The relationship between art and science can be symbiotic and making connections between these philosophically opposing paradigms has the power to foster both inspiration and innovation. Approaching the challenge of engaging seldom-heard voices creatively and employing arts-based methods to develop shared understanding and productive collaborations continues to develop traction throughout health services and health research [17]. Creative methods offer one possible solution to the challenges of engaging with marginalized people in PI, such as those who rely on AAC.

Designers and design researchers have a long track record of involving end users in the design of products, systems, and services using creative and practical methods to enable participation and disrupt the researcher/participant hegemony. A group of methods bracketed under the umbrella term ‘participatory design’ is about involving and collaborating with end-users throughout the design process to generate an outcome that reflects a shared understanding [18]. Outcomes from participatory design are commonly information technologies, but the principles of participatory design can be expanded to develop artefacts, processes, and systems. Previous examples of using creative methods inspired by participatory design principles in health research include the use of representational artefacts in service design [19] and Lego®-based play in team building [20]. Techniques which have been used to support people who have communication difficulties to participate in research include the use of photo diaries, story grids and tangible avatars in co-developing design languages [21].

The general principles of participatory design can be adopted to structure the development of shared understanding between researcher and PI representatives which can, in turn, help to redistribute power within their relationship [20]. Participatory design principles and creative co-design techniques offer health researchers the frameworks and methods through which inclusionary and truly collaborative research involvement with marginalized groups can be achieved. The flexibility and interpretivism inherent in such creative research paradigms allow for the development of bespoke methods for involvement that meet the needs of specific patient groups.

## Methods

### The research project

The Unspoken Voices Project is a National Institute for Health Research (NIHR) funded doctoral research project. The aim of the Unspoken Voices Project is to develop the conceptual framework for a PROM for people who use AAC. A group of individuals who have communication difficulties and use AAC were invited to join a PI group to provide input on developing accessible processes for items 1–5 of the PI framework for PROM development presented by Carlton et al. [10] and modified in Table 1.

In this article, we illustrate how using creative methods inspired by participatory design principles have improved the authenticity and impact of PI which has informed the development and implementation of The Unspoken Voices Project.

### The PI Group

Clinical and support staff from AAC service representatives within the project team (KB, SJ) identified individuals with lived experience of AAC who had both experience and insight that would benefit the project. The group facilitator (KB) invited these individuals to join the PI group at the start of the project in 2017. Those invited had a broadly representative range of underlying medical conditions, age groups and communication methods. The facilitator invited two females to join the group and they declined to participate. The PI group membership consists of seven expert AAC users, five of whom have communication partners who accompany them to the meetings and facilitate their involvement in certain tasks and activities (see Table 2 for a summary of the group member characteristics).

**Table 2:** Characteristics of PI Group Members

Underlying medical condition	Gender	Age category	Type of AAC used	Communication method	Communication partner/facilitator during meetings
Cerebral palsy	M	40–60	Tablet computer (Grid Pad^a^); Grid 3 software^a^; switch access (joystick switch mounted on wheelchair)	AAC device; Makaton sign; speech - single words/short sentences (slurred speech, understood by familiar listeners)	Life partner attended all meetings and interpreted speech and sign on behalf of group member when these were used
Head injury	M	18–40	Tablet computer (Grid Pad^a^); Grid 3 software^a^; touch screen access	AAC device; thumbs up/down for yes/no; no speech	Speech and language therapist or key worker support: repeated and simplified instructions when necessary, encouraging group member to find appropriate vocabulary on screen, provided physical access to resources; informed group of when a message had been constructed on AAC.
Cerebral palsy	M	18–40	Tablet computer (Mobi 2^b^); Mind Express software^c^; touch screen access	AAC device; head nod/ shake for yes/no; no speech	Speech and language therapist or key worker support: encouraged group member to find appropriate vocabulary on screen, supported physical access to resources
Cerebral palsy	M	40–60	Tablet computer (Grid Pad^a^); Grid 3 software^a^; eye-gaze access (eye-gaze camera integrated into computer)	AAC device; eye movement for yes/no; no speech	Personal assistant: informed the group when group member was constructing a message on AAC, supported with physical access resources
Primary Lateral Sclerosis	M	60–80	Hand-held, dedicated communication aid device (Lightwriter Swift^d^); Direct, manual access	Speech – uses full sentences (often quiet and/or slurred); AAC device when speech is not understood for some single words and to provide information such as introductions	Group facilitator would sometimes repeat what group member was saying if he was not understood/heard by the rest of the group
Stroke	M	60–80	iPad computer; Predictable software^e^; direct, manual access	AAC device; gesture (thumbs up/ thumbs down, head shake, shrug); no speech	Group facilitator would identify when messages were being constructed on AAC and create space in the meeting for the message to be produced synthetically and heard
Head injury	M	18–40	Tablet computer (Tobii i15^f^); Grid 3 software^a^; eye-gaze access (eye-gaze camera integrated into computer)	AAC device; smile for ‘yes’, head shake for ‘no’; no speech	Speech and language therapist or key worker support: repeated and simplified instructions when necessary, encouraging group member to find appropriate vocabulary on screen, provided physical access to resources; informed group of when a message had been constructed on AAC.

Key to manufacturer details for types of AAC: ^a^Smartbox (thinksmartbox.com); ^b^Techess (techess.co.uk); ^c^Mind Express (mindexpress.be); ^d^Abilia (abilia.com); ^e^Therapy Box (therapy-box.co.uk); ^f^Tobii (tobii.com)

### Study design

This article presents the PI group for the Unspoken Voices Project as a longitudinal case study. The facilitator introduced creative methods in a stepwise way during a series of seven groups held over a period of 28 months. The aims of employing creative methods were: a) to enable the group members to fully engage in the process of providing valuable input to the project, and b) to understand their individual roles within the project. Data presented within this case study came from meeting minutes, video recordings, written and verbal feedback from group members, and from images representing PI contributions created by a graphic artist (SS). Following each meeting, the group facilitator (KB) took a step back to evaluate the methods and looked at the extent to which those methods facilitated group member involvement. The facilitator consequently either further developed and refined or removed a method from the resources.

Baseline data for this case study come from Meeting 1 where the facilitator set up the meeting based on guidance to researchers developed by INVOLVE [21]. During this meeting, the facilitator paid particular attention to identifying an accessible venue, sending out material electronically in advance, making the agenda succinct, and adapting written materials to include short phrases, common words, and with the addition of some simple diagrams, in line with existing guidance. During the initial group meeting, there was little interaction between group members. One group member commented that they did not understand much of the language and terminology used and they could not interact with the materials provided stating that they were too “text-heavy”. These observations and comments act as baseline data for the case study so that comparisons can be made with methods employed during subsequent meetings.

The intention of employing creative methods was initially to shift the focus of the group from information exchange between group members and facilitator, based on specific topics towards a more inclusive discussion where ideas could be explored through the process of engaging in an activity. The facilitator recognised the ability of group members to reflect on and confidently describe their role and she considered this a strong indication that a shared understanding between herself and the group had been reached and hence a redistribution of power had been achieved. Therefore, the support project team (KB, KS, CC, GJ, SJ) agreed that the group had established meaningful collaboration at a stage at which examples of significant involvement could be identified and group members could describe their role and purpose within the project.

### Participatory design methods

Participatory design methods, described by Simonsen and Robertson [18] in terms of enacting, seeing and doing, provided a framework for the stepwise introduction of creative methods in the group. The facilitator included an additional stage, ‘reflecting’, to gauge individual responses to the project and enable group members to reflect on their roles within it.

#### Enacting: audio-visual media (meeting 2 onwards)

**Fig. 1:**
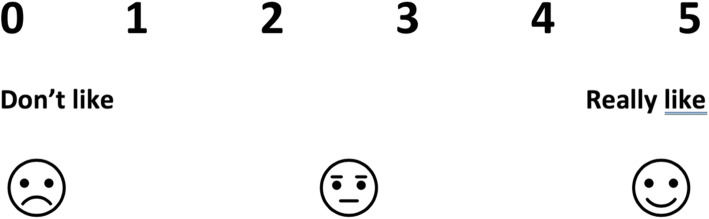
An Example of a Visual Analogue Rating Scal

The facilitator produced audio-visual agendas and minutes using PowerPoint™ with voice-over and uploaded to YouTube™ (for an example see: https://youtu.be/exw3HH4f4mE). The facilitator sent a link to group members to view the film. The facilitator produced films of recruitment materials in a similar way and shared these with the group during a meeting. The PI group provided feedback about the pictures and content of films, rating them using scales such as the one presented in Fig. 1 or Lego® blocks (the greater the number of blocks signified higher levels of approval). The facilitator asked group members, with the support of their communication partner where physical difficulties impeded reliable movement, to rate their opinion using the scale or the blocks. Where communication partners supported them in the activity, the facilitator checked back on the responses with the experts themselves, using closed yes/no questions.

#### Seeing: use of imagery (meeting 3 onwards)

**Fig. 2:**
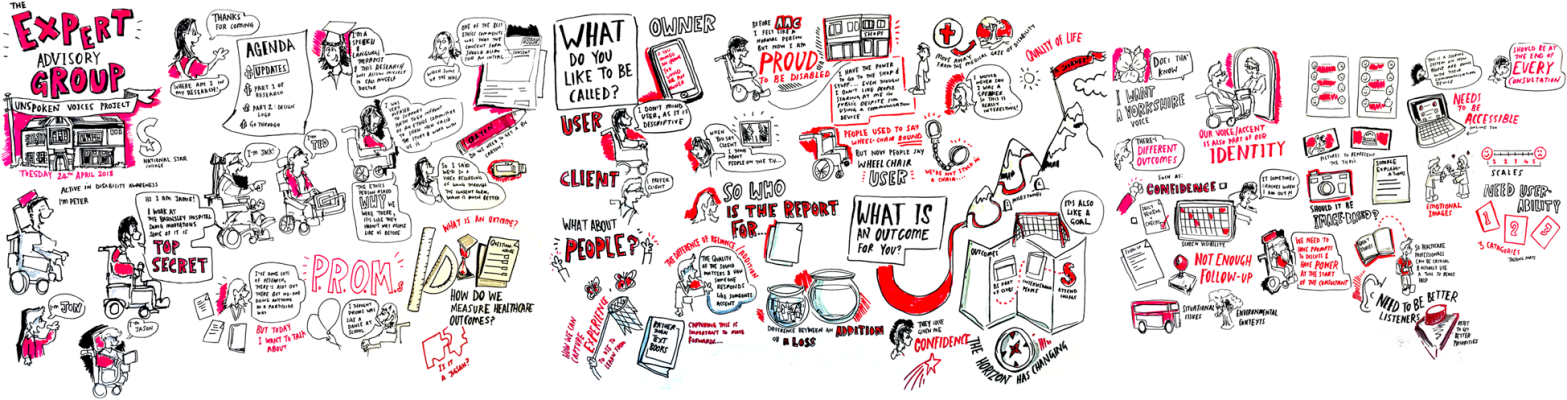
Graphically Illustrated Minutes Produced by SS (Reproduced courtesy of Smizz©)

The facilitator invited an artist (SS) to graphically illustrate the discussion that took place within group meetings. Figure 2 presents an example of the graphic minutes. The artist took photographs of the illustrations at the end of the meeting and the facilitator cut and pasted sections of the digital images into PowerPoint™ to create the film version of the minutes of the meeting.

The group also used images to represent discussion topics as a method for supporting thier involvement through *seeing*. The facilitator identified a range of images by searching keys words in image libraries in internet search engines in advance of the meetings, which everyone used to represent discussion topics. The images provided stimuli for the group discussions and helped the group to explore the meanings that individual members ascribe to a particular image.

#### Doing: talking Mats™ (meeting 4 onwards)

**Fig. 3:**
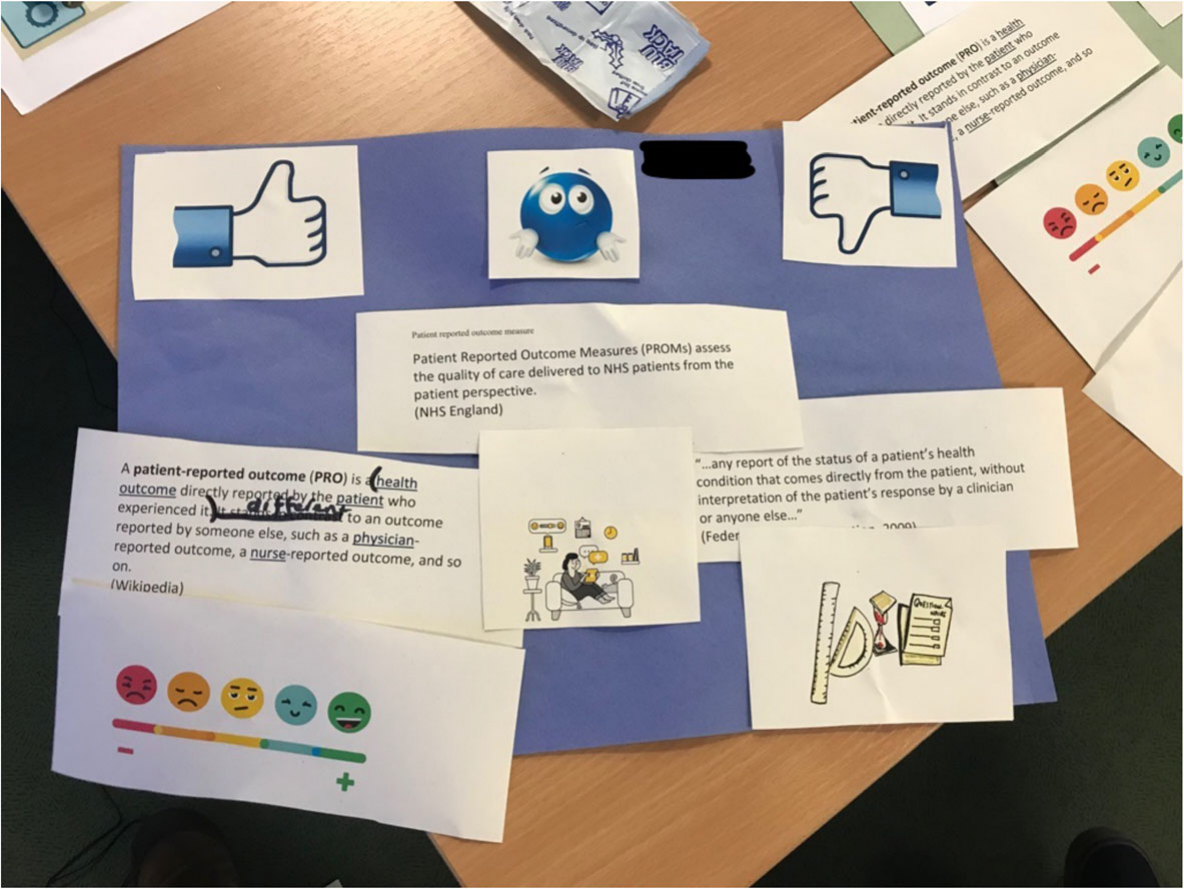
Example of a Talking Mat Activity

Talking Mats™ is a collaboratively produced, picture or text-based tool that therapists and researchers have used to gather opinions and feedback from people who have communication difficulties, in both research and service settings [22]. During Talking Mats™ mediated interactions, all facilitators encouraged, supported or helped group members to arrange a set of topic-specific words, pictures or symbols onto a mat across a three-point scale: positive, neutral, negative. The group facilitator provided a range of prompts (words, phrases and images), pertinent to the topic being explored and asked group members to rate them on a Talking Mat™ (Fig. 3).

#### Reflecting: object metaphors (meetings 6 and 7)

In order to encourage group members to reflect on the project, the facilitator presented objects to support the generation of metaphors. The facilitator presented a range of everyday objects e.g. toys, craft materials, stationery, keys etc., and asked an open question e.g. “What does being involved in the project mean to you?”, “What is your role in the project?”. Group members selected an object which represented their response to the question. If they struggled to pick up an object due to physical difficulties, they used their AAC device to make a selection and describe why they had chosen it or used their communication partner to support their selection.

## Results

The creative methods the group employed during the PI meetings for the Unspoken Voices Project enabled them to engage in providing input to a number of areas of the research project. These methods also provided them space for contemplation and reflection to help them to understand, not only the purpose of the project but also their role within it. The stepwise introduction of methods, in terms of enacting, seeing, doing and reflecting allowed group members the opportunity to practise using each method and provide feedback on it.

At the end of Meeting 1 (the baseline), the group considered the meeting too wordy and group members reported that they did not understand key concepts concerned with the project. By Meeting 7 they had been able to provide valuable perspectives and ideas to improve the accessibility of the project. They were also able to use metaphors to describe their roles within in the project and in relation to other group members and members of the research team. Specific textual and visual data and vignettes taken from group meeting minutes are presented below to exemplify these developments.

### Enacting

The feedback from the group about the audio-visual agendas and minutes was overwhelmingly positive:

“I thought that the video content and presentation were excellent. Really genuinely. I thought it was a very fair summary of the meeting. Personally, I can cope with written material but if others favour the video and you’re willing to put in the effort, then I’m sure it will be valuable for third parties as well” (Group member, Meeting 2).

After engaging in a rating activity, members of the PI group were able to reflect on the impact that the audio-visual materials had on them and, therefore, how the recruitment materials may affect potential participants to the project: “Really clear without being patronising” (Group member, Meeting 2); “How you talk in that video was brilliant. I wish everybody who works with people who use AAC or whatever was so clear” (Group member, Meeting 2). They provided feedback using visual analogue scales to further refine the recruitment materials for the project. Use of the Lego® was less successful as some group members’ physical impairments limited the extent to which they could interact with the blocks.

During this meeting, the group also developed a shared understanding about the importance of using accessible recruitment materials. This collective meaning-making empowered a representative of the group to attend the NHS research ethics committee meeting, alongside members of the research team (KB, KS), to justify why audio-visual resources were so critical for use during recruitment to this project.

### Seeing

The use of images during meetings helped to focus group discussions and created space within meetings for group members to construct responses on their AAC devices when necessary.

The reflections that they shared about their individual interpretations of the images provided valuable insight and enabled discussion between group members. For example, in one meeting, several group members preferred a picture of a staircase to represent the term ‘outcomes’ – implying that accessing AAC resulted in positive and progressive outcomes; however, for one individual, who has primary lateral sclerosis (a degenerative condition), using AAC was not considered a positive outcome; to him, AAC represented deterioration in his speech and therefore a progression of his illness.

**Fig. 4:**
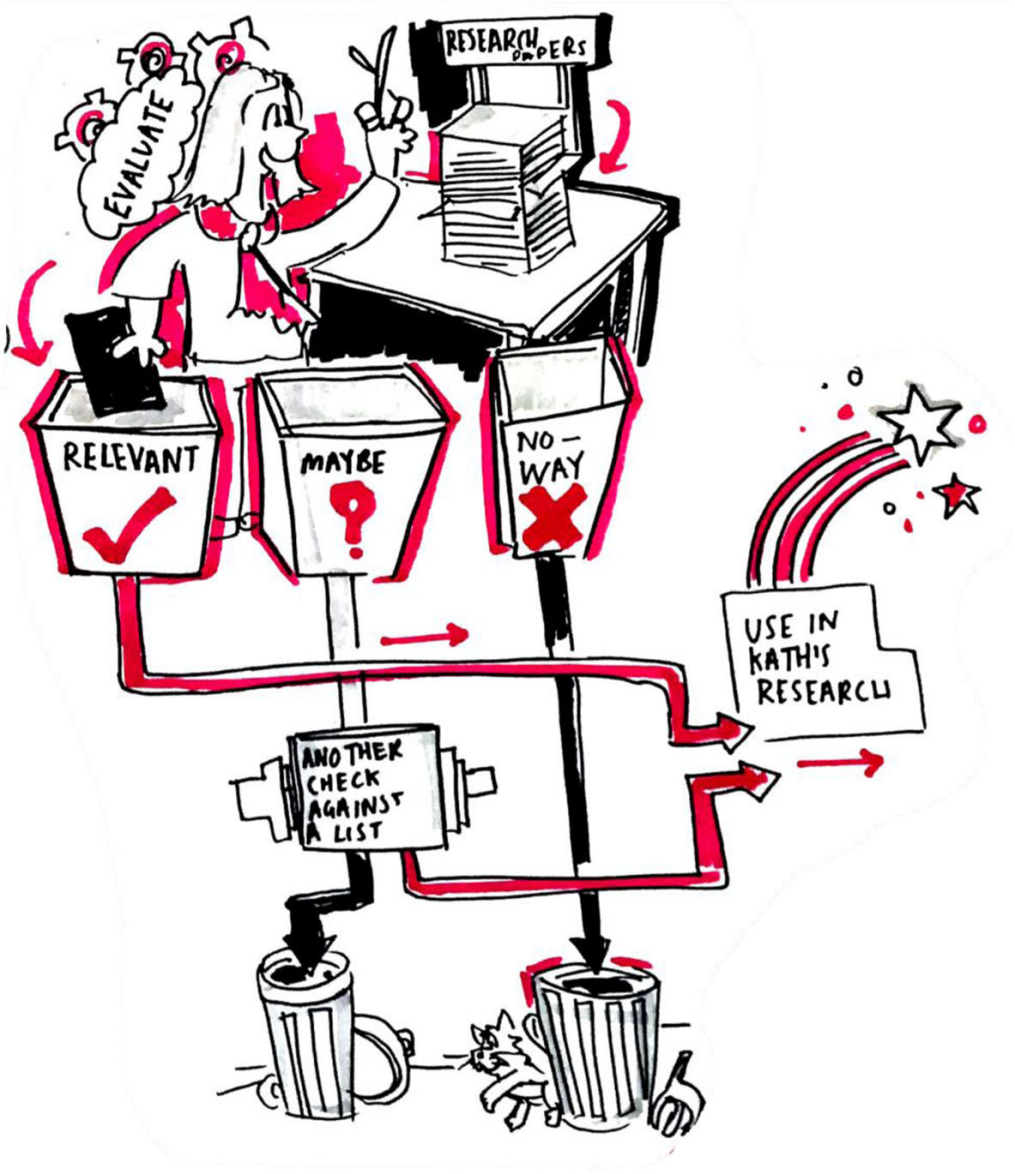
Illustration Described by a Group Member and Created by SS to Represent the Term ‘Systematic Review’ (Reproduced courtesy of Smizz©)

The artist’s visual interpretation of the activities and discussion within Meetings 3–6 helped group members to engage with some of the more abstract concepts and unfamiliar terminology related to research. Changing the emphasis of communication from speaking and words towards visual media encouraged the creation and sharing of meaning through imagery. Following a discussion about research terminology, one group member was able to describe an image that he felt represented the term ‘systematic review’ and the artist then recreated it in a graphic, presented in Fig. 4.

Visual images have implicit meanings which can be specific to an individual; sharing these meanings enabled the discussion to flow between group members rather than remain in the control of the facilitator. Communication partners were also able to participate by responding to the images and added their perspectives to the discussion within the group. They provided additional interpretations to the group’s contributions which the facilitator was able to check back with members. The artist’s representation of these discussions, as documented in the graphic minutes, provided the opportunity for further exploration of meaning by a wider audience as some of the illustrations drawn by the artist have subsequently been incorporated into dissemination materials and presentations. The illustrations have been used in conference presentations to directly represent the group members and their contributions when sharing outputs from the research study. The use of cartoon-graphics to represent group members maintains their anonymity, at the same time as personifying them and their roles within the project. Two group members planned and executed a platform presentation at a national conference by incorporating the artist’s graphics with their own pre-prepared synthetic speech voice output, stored in their AAC devices.

### Doing

**Fig. 5:**
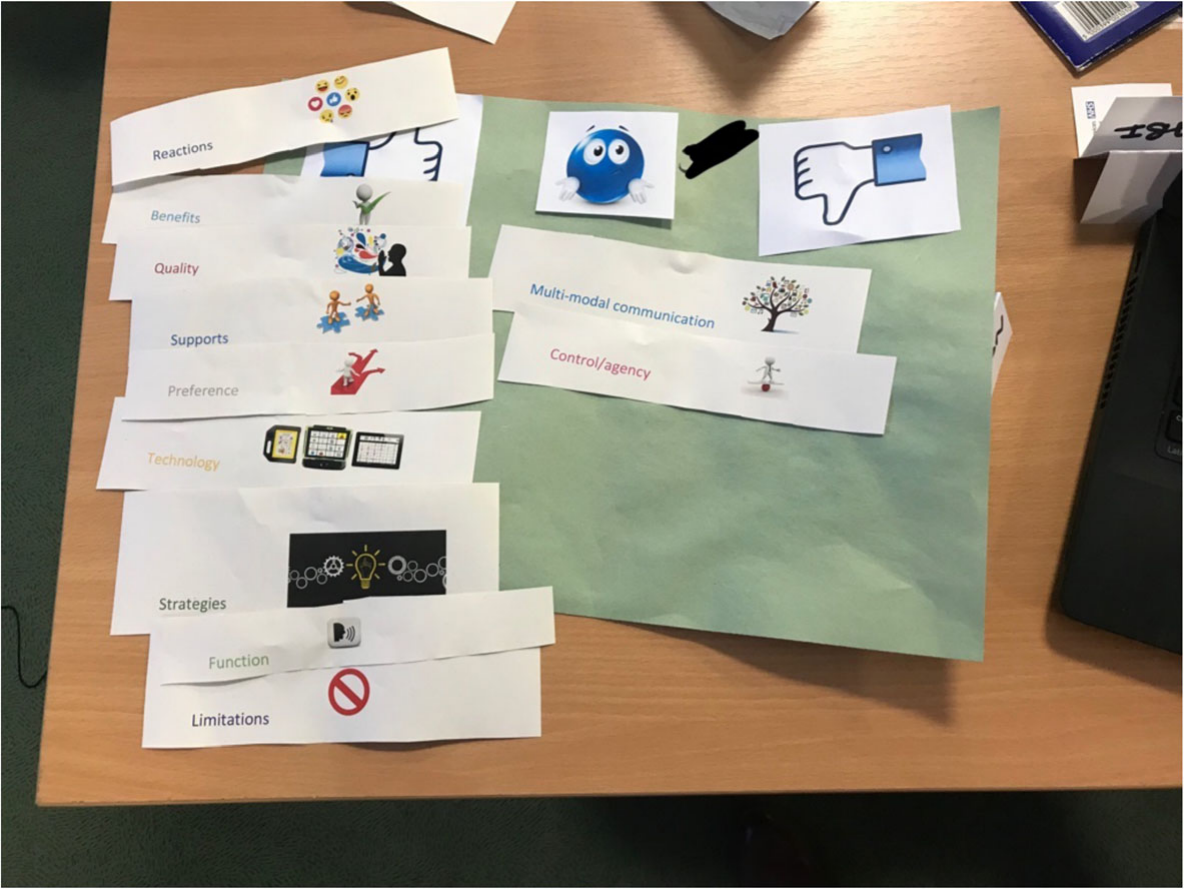
Talking Mat of Triangulation of Themes Generated from a Systematic Literature Review

The group used Talking Mats™ in Meeting 4 to develop definitions of unfamiliar terminology such as ‘PROM’, ‘systematic review’ and ‘synthesis’. During the resulting discussion the group generated of a set of agreed definitions which were then used to produce an accessible summary of a systematic literature review. Talking Mats™ was also employed in Meeting 5 to support them to analyse data from a systematic literature review. An example of the Talking Mat produced can be seen in Fig. 5. This activity, along with the subsequent discussion, illuminated additional themes which members agreed were pertinent to the review and which had been previously overlooked by the academic team.

Using visually supported activities rather than language mediated discussions enabled the members to co-construct a shared understanding of the review themes. This then facilitated group members to engage in conversations, using a range of communication strategies, about their own experiences of using AAC in a supported and enabling environment.

### Reflecting

**Fig. 6:**
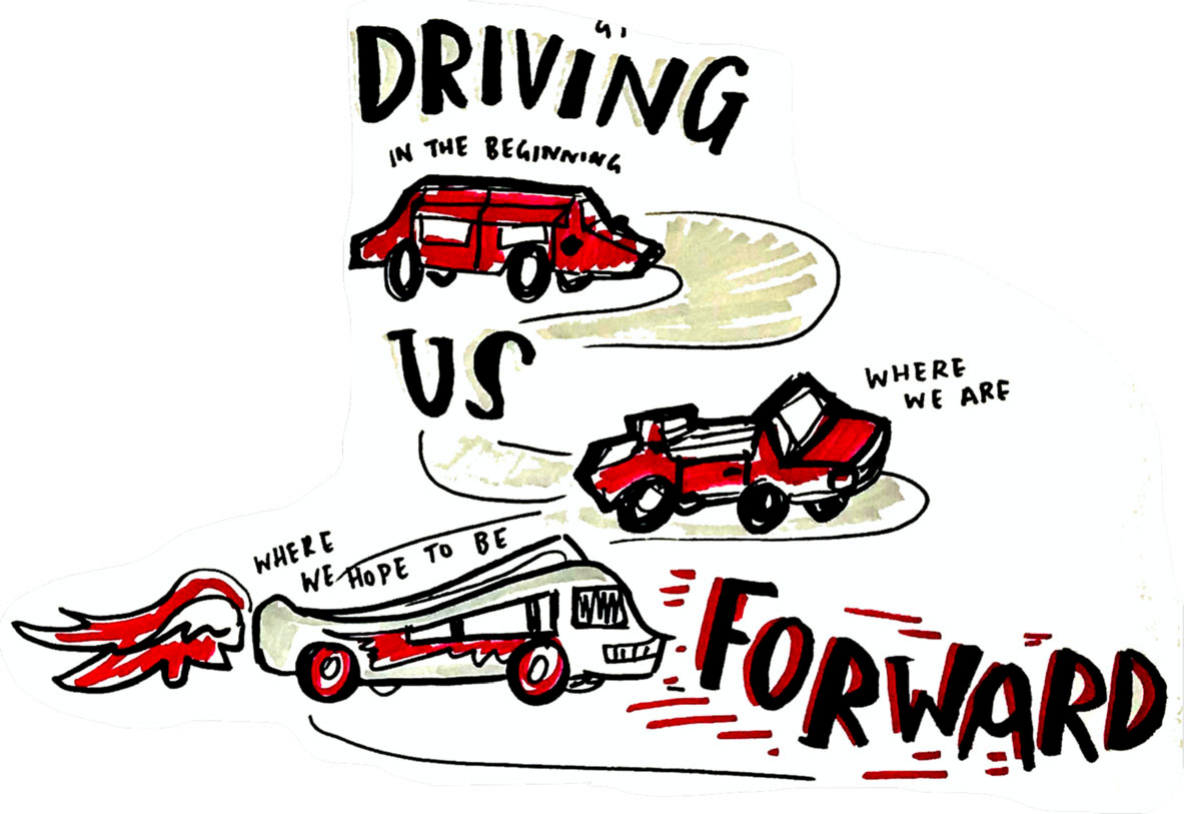
Graphically Illustrated Example of an Evolving Metaphor (Reproduced courtesy of Smizz©)

During activities in Meetings 6 and 7 which explored objects as metaphors, group members described being initially drawn to objects because they were attracted to it on some aesthetic level, for example, colourful pompoms or a toy car. The process of describing their choice led to them creating a metaphor and by describing the metaphor further discussion within the group ensued. One group member initially selected a drab, toy estate car which he felt represented the group as ‘driving forward’ change. He then chose a faster sports car, and evolved the metaphor into the car representing the project. Finally, he chose a fiery race car to represent where he saw the group going in future. The artist produced an illustration of this metaphor which the reader can see in Fig. 6.

In Meeting 7, group members arranged a selection of toys on table to represent the project and their relationship to it. They collectively chose a large vehicle (wooden campervan) to represent the project and then selected different toys and placed them in relation to this vehicle. One group member was a passenger in the vehicle but described himself as the ‘navigator’, another was overseeing the project from a helicopter that circled above the vehicle and kept it on track. The group agreed that a Duplo® propeller represented the group as whole and the impact that it was having on the Unspoken Voices Project.

The use of the activity involving objects enabled group members to reflect on their choices and to create their own metaphors. The metaphors provided the facilitator with an insight into the personal interpretations of each individual on the question posed.

## Discussion

The aim of this article is to illustrate how the use of creative methods, inspired by participatory design, facilitated the successful involvement of people who have communication difficulties and who use AAC in PI. The group has inspired discussion within the academic project team about the epistemology for the research project which helped us to challenge the traditional paradigmatic boundaries of method. The PI group also provide a mirror against which the group facilitator (KB) has reflected on her positionality: her background as a clinician (speech and language therapist), her development as an academic, and how she should, and can, ultimately assimilate these roles in future. Most importantly, group members self-report pleasure in being provided with the opportunity for personal growth that, anecdotally, is all too infrequently afforded to those who live with complex disabilities.

### Creativity and accessibility

PI for PROM development should ensure that research processes take into account the specific needs and perspectives of participants and end-users and truly collaborative PI is one mechanism for ensuring that the patient voice remains at the heart of PROM development projects [10].

The creative methods described in this article evolved and grew iteratively over a period of 28 months and 7 meetings. They represent examples of how the use of methods that are not wholly dependent on spoken language successfully facilitated the inclusion of PI group members with communication difficulties who use AAC. The greater role that the PI group has been able to play in the project, as a result of using these emancipatory methods, has had a significant impact on the research. Their roles within the project have shifted beyond what INVOLVE consider ‘consultation’ to being ‘active and collaborative members of the research project team’ ([6], p21–22).

The methods described here could be applied in other research projects with people for whom traditional PI forums, such as focus groups, are inaccessible. Creativity has applicability beyond people whose communication is non-verbal, as is being recognized in many areas of healthcare and health research [17, 20, 21]. People can feel disenfranchised from PI because of a range of social, cultural and linguistic differences that ultimately manifest as barriers to inclusion. The onus is on research teams to identify and breakdown these barriers in order to develop outcome tools that reflect the diversity of our populations. The participatory design framework presented in this article adds to the growing body of literature that is developing traction in PI and emancipatory health-research methods.

### Method

Although much has been learned during the Unspoken Voices Project about establishing a productive PI group, discussion amongst the project team concerning method continues to evolve as the project progresses. The methods described in this article specifically, and the principles of participatory design more generally, have informed not just the running of the PI group but also the theoretical lens through which the project can be viewed. Developing a PROM requires the use of rigorous methods to ensure that the tools developed for use in clinical practice are robust [7]. There are several well-documented quantitative methods that validate the psychometric properties of PROM tools [7]. It is also acknowledged that patients play a significant role in PROM development [9, 10] and PI is one mechanism that can enable patient input to the research cycle. The creative methods described in this article have resulted in authentic collaboration with the PI group and the impact has been the development of a more accessible research project that will create a PROM. The impact that some of these methods have had on the level of involvement afforded to people who have significant communication difficulties highlights the potential emancipation that creativity and interpretivism can offer to seldom heard or unspoken voices.

Looking beyond the boundaries of traditional health-research methods towards more creative disciplines can expand the repertoire of tools available to research teams and can achieve greater collaborative research [19–21]. The use of creative methods in the PI group enabled the research team to reach beyond tokenistic consultation, achieve valuable involvement and provides further support for strengthening the presence of both art and science in healthcare and health research*.*

### Positionality

Developing a career as a clinical academic can present some paradoxical challenges. Professional training and experience can lead a clinical academic researcher to bring particular skills but also biases to their research project. As a speech and language therapist with several years of experience in running therapy groups, the group facilitator (KB) had initially been confident in her ability to manage the PI group meetings and to use her clinical skills to facilitate the involvement of group members despite their communication difficulties. However, the nature of the clinician-patient hegemony that exists in healthcare provision is in many ways subconscious to an attentive and reflective clinician and although unintentional, there were probably elements of this dynamic at play during the initial, unsuccessful group meeting. In attempting to adhere to existing PI frameworks and guidelines, KB, on reflection, also overlooked the core clinical skills that may have been of value in that group meeting such as focusing on implementing supportive communication strategies.

It is important to acknowledge the critical role that the facilitator plays within a PI group in establishing the overall structure of the group and in creating the relationships that will ultimately drive it forward, as well as in selecting and implementing appropriate methods to realise both these elements. Employing participatory design principles provided both the framework and the license for KB to draw together her professional background with the aims and objectives of the group, to create a more facilitative environment. Synthesizing professional skill and experience with learning how to be a researcher is a pivotal part of the development of clinical academics and such interdisciplinary practice and reflection could prove to be of value for personal and professional development in other areas of academia.

### Perceptions of PI

Finally, but perhaps most importantly, establishing a group which facilitated constructive involvement and brought equipoise to the relationship between the facilitator and group members also created an opportunity for participants to consider what being a PI expert for this project meant to them. They were provided space to explore not just what they brought to the project but also how they viewed their role and what they got out of their involvement. PI contributions are often remunerated by way of cash payments or vouchers [6]. Group members for this project were provided with an accessible form to complete in which they could select or indicate their preference for remuneration and acknowledgement of their contribution to the project. They completed the forms independently, or with support from their communication partners; none of the group members for this project opted for a financial payment. One group member who contributed to this article opted out of authorship, preferring the role of mentor and guide. Another two group members were glad of the opportunity to present at conferences but felt less inclined to support with written project outputs. A fourth group member, who has a doctorate, was keen for acknowledgement in project outputs, in particular, the final thesis for the research. These responses provoke questions about how the research community consider the impact that PI has on the individuals involved. Is their time and participation best accounted for in monetary terms or does their personal view of their role within the project warrant deeper consideration in terms of the impact it has on a sense of social responsibility, self-identity, or something else? Checklists allow people to make choices about how they want to be involved but exploring roles through metaphors opens up the door for conversations about *why* people want to get involved. A more considered appreciation of the meaning of PI roles for individuals within the group, perhaps using creative and interpretivist methods, may help researchers both manage expectations and create opportunities for PI development both in terms of the group as a whole and each individual member.

### Areas for future development

This paper presents the experiences of a single PI group for a specific research project. The results represent possible next steps toward improving the mechanisms for involvement by people who have communication difficulties and who use AAC in research. Building on the work of previous projects [15, 16], we have attended to the particular methods that can be used to support involvement and collaboration with these seldom heard voices but also accept that there is more work to be done in this field.

The PI group consisted of people with a range of educational backgrounds and ages, who had experience of different medical diagnoses and communication difficulties. Representation could be improved by the inclusion of females and people from black and minority ethnic backgrounds in the group. There were several logistical challenges in arranging group meetings including travel times, educational term dates and the resource required to plan and implement the meetings, that resulted in the group convening 2–3 times per year. The inability to meet more frequently may have had an impact on the continuity of the relationships that were developed within the group.

Interactions with people who have communication difficulties and who use AAC is frequently mediated by a communication partner and the co-construction of meaning within interactions is shaped by the shared contextual understanding that underpins this relationship [14]. We have highlighted the role of the communication partner in supporting communication and provided some examples of where their input and contributions supported group member’s involvement. There is a need for a deeper understanding of the nature of these interactions and specifically in the role that communication partners play within the dialogue with people who use AAC which would further inform the interpretations that are drawn through using creative methods.

Finally, we would like to see more reporting of the methods used in other PI groups who have achieved some success in developing collaborative relationships and productive involvement of people with seldom heard voices. We appeal to fellow research teams to be confident and generous in sharing their experiences so that we can continue to nurture and grow opportunities for greater involvement in research.

## Conclusion

The role of participatory design methods through engaging principles of enacting, seeing, doing, as well as reflecting, used in the PI group for the Unspoken Voices Project is presented as a case study. Using creative methods has provided mechanism for genuine involvement with people who have communication difficulties and who use AAC resulting in both a more accessible research process and impactful dissemination of results. Creativity has an important role to play in the emancipation of public involvement to a range of seldom heard voices and should be added to the methods employed in building collaborative relationships between academics and other members of project teams. Running PI groups creatively can improve the accessibility of research, provoke researchers to shift their thinking from their traditional paradigms and provide space and opportunity for PI members to more deeply appraise the value they attribute to their contribution which may support them on their own personal development journey.

## Data Availability

The dataset supporting the conclusions of this article is included within the article and its additional files.

## Abbreviations

PI: Public involvement
PROM: Patient-reported outcome measure
AAC: Augmentative and alternative communication
PLS/MND: Primary lateral sclerosis/motor neuron disease
